# Transcriptome Profiling Reveals That the African Swine Fever Virus C315R Exploits the IL-6 STAT3 Signaling Axis to Facilitate Virus Replication

**DOI:** 10.3390/v17030309

**Published:** 2025-02-24

**Authors:** Shuxian Geng, Zhonghui Zhang, Jie Fan, Hualin Sun, Jifei Yang, Jianxun Luo, Guiquan Guan, Hong Yin, Qiaoying Zeng, Qingli Niu

**Affiliations:** 1 College of Veterinary Medicine, Gansu Agricultural University, Lanzhou 730070, China; g2928520057@outlook.com; 2 State Key Laboratory for Animal Disease Control and Prevention, College of Veterinary Medicine, Lanzhou Veterinary Research Institute, Chinese Academy of Agricultural Sciences, Lanzhou University, Lanzhou 730046, China; zhangzhonghui6@139.com (Z.Z.); 82101221639@caas.cn (H.S.); yangjifei@caas.cn (J.Y.); luojianxun@caas.cn (J.L.); guanguiquan@caas.cn (G.G.); yinhong@caas.cn (H.Y.); 3 African Swine Fever Regional Laboratory of China (Lanzhou), Gansu Province Research Center for Basic Disciplines of Pathogen Biology, Lanzhou 730046, China; 4 College of Medicine, Northwest Minzu University, Lanzhou 730030, China; wsbnzybaooab@126.com; 5 Jiangsu Co-Innovation Center for the Prevention and Control of Important Animal Infectious Disease and Zoonosis, Yangzhou University, Yangzhou 225009, China

**Keywords:** African swine fever virus, C315R, RNA sequencing, interleukin-6, signal transducers and activators of transcription-3

## Abstract

African swine fever (ASF) is an acute and highly contagious disease that has caused great losses in the past years. It is caused by African swine fever virus (ASFV), which is a large DNA virus encoding about 165 genes. It has been shown that the purified extracellular ASFV is internalized by both constitutive macropinocytosis and clathrin-mediated endocytosis, and the virus utilizes apoptotic bodies for infection and cell cell transmission. The ASFV-encoded RNA polymerase subunit C315R is thought to play an important role in ASFV replication and transcription. However, its involvement in ASFV infection, particularly in host response, remains only partially understood. In this study, the role of C315R in enhancing ASFV replication was investigated through RNA-Seq transcriptomic analysis, which was based on 3D4/21 cells transfected the plasmid expressing HA-tagged C315R or the empty vector. Our findings revealed that C315R significantly upregulates the expression of inflammatory mediators with a particular emphasis on IL-6. The most differentially expressed genes (DEGs) were predominantly associated with the TNF, IL-17, MAPK, and JAK STAT signaling pathways. RNA-seq results were validated through RT-PCR. Subsequently, we observed that ASFV infection increases IL-6 expression and STAT3 phosphorylation, which is regulated by the ASFV C315R protein. Notably, inhibiting STAT3 phosphorylation with specific inhibitors suppressed ASFV replication. In conclusion, our study demonstrates that the ASFV C315R protein actives STAT3 phosphorylation through promoting the transcription of IL-6 to facilitate virus replication. These findings highlight C315R as a positive regulator in the IL-6 STAT3 signaling axis during ASFV infection.

## 1. Introduction

African swine fever (ASF) is an acute and highly contagious disease caused by African swine fever virus (ASFV), which is primarily transmitted between *Sus* *scrofa* (domestic and wild pigs) and warthogs (*Phacochoerus africanus*) via soft ticks of the genus *Ornithodoros*. ASF was first reported in Kenya in 1921 by R.E. Montgomery, who described its devastating effects on domestic pigs, with mortality rates approaching 100% [1]. The first European outbreak occurred in Portugal in 1957, which is likely due to feeding pigs contaminated food waste [2]. The disease emerged in China in 2018 and subsequently spread to South Asia, East Asia, Southeast Asia, and Oceania [3]. Based on clinical symptoms, ASF is classified into three forms: per-acute, acute, sub-acute, and chronic [4]. The primary clinical manifestations include depression, widespread hemorrhaging in internal organs, and high fever. Infections result in high mortality rates among domestic pigs and wild boars, causing severe economic losses to the global pig industry [3,4].

ASFV belongs to the nucleocytoplasmic large DNA virus (NCLDV) family and possesses a complex genome that varies in size from 170–190 kb [5], which encodes 150–200 viral proteins that are involved in diverse functions, including DNA replication, transcription, RNA modification, and processes regulating host cell activity, ultimately facilitating viral immune evasion [6]. The ASFV genes can be categorized into four groups: immediate-early, early, middle, and late genes, which are expressed in a tightly regulated cascade [5,7]. Immediate-early and early genes are transcribed before DNA replication, whereas middle and late genes are expressed post-replication [5]. ASFV utilizes a relatively independent replication and transcription system. DNA replication predominantly occurs in viral factories (VFs) near the perinuclear region with an initial replication phase in the cell nucleus [8]. Entry into the host cell is mediated by phagocytosis, macropinocytosis and clathrin-mediated endocytosis, following which the virus targets early endosomes [9]. Following viral uncoating, the inner capsid membrane fuses with late endosomes, releasing the viral genome and initiating intranuclear replication [10]. This early replication phase, which peaks around 6 h post infection (hpi), transitions to cytoplasmic replication after 12 hpi [11].

Some ASFV genes, such as *A104R* and *78R*, are crucial for DNA replication, with A104R localized to replication sites and K78R accumulating in the nucleus during late-stage replication [12,13]. Additional replication-associated genes, including *E301R, G1122R, P1192R, E165R, F1055L*, and *B962L*, encode proteins such as proliferating cell nuclear antigen (PCNA)-like proteins, DNA polymerase family B, DNA topoisomerase II, dUTPase, and helicase [5,14]. Several ASFV proteins, such as pA104R and pP1192R, also exhibit DNA supercoiling activity [5,12]. Another notable gene, C315R, encodes a transcription factor resembling TFIIB (pC315R), which collaborates with the ASFV RNA polymerase (RNAP) to form a transcription initiation complex [15]. The ASFV core RNAP is capable of autonomously recognizing poly-T terminators, independent of host termination factors, which underscores the virus’s ability for self-regulated transcription [14]. Considering the crucial function of C315R in viral transcription, exploring possible interactions between C315R and host factors could offer a deeper understanding of ASFV host interactions that extend beyond mere transcriptional control. The identification of these host factors might uncover new ways in which ASFV adjusts the host environment to promote infection and replication. Overall, the C315R gene is essential for viral DNA transcription and the replication cycle, highlighting its pivotal role in ASFV pathogenesis [16,17,18].

The JAK STAT signaling pathway is crucial in gene transcription and viral replication regulation by modulating the host immune response [19]. Upon viral infection, cytokines or signaling molecules activate Janus kinases (JAKs), which cause the phosphorylation of Signal Transducers and Activators of Transcription (STATs) [20]. Activated STATs migrate to the nucleus to begin the expression of antiviral genes [21]. While essential for immune defense, some viruses exploit the JAK STAT signaling pathway to enhance replication [20]. For instance, the influenza A virus NS1 protein interferes with STAT1 activation, aiding viral replication [22]. HIV activates STAT1 and STAT3 but evades the pathway by altering cytokine signaling and impairing JAK STAT function, enabling persistent infection [21,23]. Herpes Simplex Virus (HSV) similarly manipulates the JAK STAT pathway, inhibiting STAT1 and STAT3 activation to evade immune defense and promote replication [22,24]. Collectively, the JAK STAT pathway plays a pivotal role in antiviral immunity; many viruses have evolved mechanisms to hijack or suppress it, highlighting its potential as a therapeutic target for antiviral treatments [25].

In this study, we explored the molecular mechanisms underlying the role of the ASFV C315R protein in modulating host cellular pathways to facilitating viral replication. Through transcriptomic profiling, we found that C315R can upregulate IL-6 expression, which in turn activates STAT3 phosphorylation, leveraging the IL-6 STAT3 signaling axis, which is a critical pathway in immune regulation and cell survival to promote ASFV replication. These results provide novel insights into the molecular interactions between ASFV and host signaling pathways, highlighting potential targets for antiviral strategies.

## 2. Materials and Methods

### 2.1. Biosafety and Ethics Statement

All experiments involving live ASFV were performed in a bio-safety level-3 (BSL-3) facility at the Lanzhou Veterinary Research Institute (LVRI), Chinese Academy of Agriculture and Sciences (CAAS), accredited by the China National Accreditation Service for Conformity Assessment (CNAS) and approved by the Ministry of Agriculture and Rural Affairs of China. The animal treatments and sample collection were approved by the Animal Ethics Committee of the LVRI, CAAS (LVRIAEC-2023-043). All animals were handled in accordance with the Animal Ethics Procedures and Guidelines of the People’s Republic of China.

### 2.2. Cells and the Virus

3D4/21 cells (ATCC CRL-2843; immortalized porcine alveolar macrophages, IPAM) were cultured in RPMI 1640 medium (Servicebio, G4535-500ML, Wuhan, China) supplemented with 10% fetal bovine serum (FBS; SORFA, SX3000, Huzhou, China) and 1% penicillin–streptomycin solution (Solarbio, P7630, Beijing, China). The cells were maintained at 37 °C in a 5% CO_2_ incubator. Red blood cells were prepared from heparin sodium-treated swine blood and stored in RPMI 1640 medium containing 10% FBS and 1% penicillin–streptomycin solution at 4 °C. The ASFV CN/SC/2019 strain was isolated, identified, and maintained in the bio-safety level 3 (BSL-3) laboratory of the Lanzhou Veterinary Research Institute (LVRI), Chinese Academy of Agricultural Sciences (CAAS). The recombinant virus ASFV-eGFP, with the insertion of only an eGFP tag, was generated from the parental strain ASFV CN/SC/2019 and maintained in LVRI-BSL-3.

### 2.3. Antibodies

A monoclonal antibody against the p30 protein was developed and stored in our laboratory. The following antibodies were procured from the corresponding companies: anti-β-tubulin rabbit antibody (Proteintech, 10068-1-AP, Rosemont, IL, USA), anti-GAPDH mouse antibody (Proteintech, 60004-1-Ig, Rosemont, IL, USA), anti-DYKDDDK-tag rabbit antibody (Abmart, R20008M, Shanghai, China), anti-HA-tag rabbit antibody (Abmart, P60025M, Shanghai, China), anti-STAT3 rabbit antibody (CST, 4904S, Danvers, MA, USA), anti-pSTAT3 (ser727) rabbit antibody (CST, 9134S, Danvers, MA, USA), anti-IL-6 rabbit antibody (BBI, D620828-0100, Shanghai, China), and anti-H2AC6-rabbit antibody (Solarbio, K110514P, Beijing, China).

### 2.4. RNA Extraction and Transcriptomic Sequencing

3D4/21 cells were seeded in 6-well plates with RPMI 1640 medium (Servicebio, G4535, Wuhan, China) supplemented with 10% FBS and 1% penicillin streptomycin solution. The cells were divided into two groups and transfected with 2 μg of either pcDNA3.1-HA or pcDNA3.1-HA-C315R for 24 h. Total RNA was then extracted using Trizol reagent (Invitrogen, 15596026, Waltham, MA, USA), and polyadenylated mRNA was selectively captured using Oligo (dT) primers (Thermo, SO131, Waltham, MA, USA). The mRNA was fragmented into 200 bp fragments using the NEB Next Magnesium RNA Fragmentation Module (NEB, E6150S, Ipswich, MA, USA) and served as templates for double-stranded cDNA (ds cDNA) synthesis using the ds cDNA Synthesis Kit (YEASEN, 13488ES08, Shanghai, China). The result dscDNA underwent end-repair, A-tailing, and ligation with indexed adapters. The final sequencing library was prepared and sequenced on the Illumina Nova Seq 6000 platform by Gene Denovo Biotechnology Co., Ltd. (Guangzhou, China). The differential expression of miRNAs and mRNAs was analyzed using DESeq2 software, v. 3.20 with thresholds set at |log₂ fold-change (FC)| > 1 and *p* < 0.05.

### 2.5. Enrichment Analysis and Protein Protein Interaction Network Analysis

Gene Ontology (GO) annotation, Kyoto Encyclopedia of Genes and Genomes (KEGG) and Reactome enrichment analysis were analyzed using data from the GO database (http://www.geneontology.org (accessed on 20 December 2024)), KEGG database (https://www.genome.jp/kegg/pathway.html (accessed on 20 December 2024)) and Reactome database (https://reactome.org (accessed on 20 December 2024)), respectively. Protein protein interaction networks were analyzed by using the STRING database (https://cn.string-db.org/ (accessed on 20 December 2024)).

### 2.6. RT-qPCR

3D4/21 cells were transfected with 2 μg of either pcDNA3.1-HA (NC control) or pcDNA3.1-HA-C315R for 24 h. Total RNA was extracted from each sample using Trizol (Invitrogen, 15596026, Waltham, MA, USA), and cDNA was synthesized using the PrimeScript™ RT Reagent Kit with gDNA Eraser (Takara, RR092S, Osaka, Japan) according to the manufacturer’s instructions. Reverse transcription PCR (RT-PCR) was performed using the Step PrimeScript RT-PCR Kit (Takara, RR064B, Osaka, Japan). Relative mRNA expression levels were calculated using the comparative cycle threshold (CT) method (2^−ΔΔCT^). The primer sequences used for viral and cellular gene amplification are listed in Table 1. Data analysis was conducted using GraphPad Prism 8.0.2 software.

### 2.7. Immunohistochemistry

Archival or fresh pig samples (blocks or formalin-fixed paraffin-embedded slides) were prospectively centrally assessed for H2A clustered histone 6 (H2AC6) expression using the immunohistochemistry (IHC). Thicks were mounted on poly-L-lysine coated slides (Wuhan, WHB-24-CS-10, China). The sections were dewaxed and boiled for 30 min in repaired work solution (Immuno, B04135, Beijing, China), and washed in distilled water. The tissues were incubated overnight with primary antibodies in the horseradish peroxidase-conjugated secondary antibody (Abbkine, A21020, Atlanta, GA, USA) for 1 h. The reaction used 5% (*w*/*v*) DAB (GlpBio, GC18494-5.1, Montclair, CA, USA) as a substrate, and all the sections were counterstained with hematoxylin. Scale bars: 20 μm.

### 2.8. Western Blotting Analysis

All cell samples were lysed in RIPA buffer (Thermo, 89900, Waltham, MA, USA) supplemented with a protease inhibitor cocktail (Thermo, 87785, Waltham, MA, USA) and incubated on ice for 2 h. The lysates were centrifuged at 12,000 rpm for 30 min at 4 °C to remove cell debris, after which we transfered the supernatant to a new tube. For Western blotting analysis, the proteins in equal amounts of cell lysates were then denatured in SDS-PAGE loading buffer for 10 min, after which they were separated by 10–12% SDS-polyacrylamide gel electrophoresis (PAGE) and transferred to nitrocellulose (NC) membranes (Merck-Millipore, HATF00010, Billerica, MA, USA). The membranes were blocked with 5% (*w*/*v*) skimmed milk for 2 h at room temperature to block nonspecific binding sites. For immunoblot analysis, the membranes were incubated overnight at 4 °C with specific primary and secondary antibodies for 1 h at room temperature. Protein bands were detected using the Crescendo ECL detection system (BIO-RAD, 1705061, Hercules, CA, USA) via chemiluminescence and autoradiography.

### 2.9. Cell Viability Assay

Cell viability was assessed with a CCK-8 kit (APExBIO, K1018-5, Houston, TX, USA) following the manufacturer’s instructions. In brief, PAMs were seeded (2 × 10^5^) in 96-well plates and cultured for 24 h before treatment with Stattic (Selleck, S7024, Houston, TX, USA) for 48 h. Subsequently, 10 µL of CCK-8 solution was added to each well, and cell viability was assessed after incubation for 2 h at 37 °C. The absorbance was measured at 450 nm, and cell viability was calculated according to the following equation: cell viability rate (%) = [(OD inhibitor − OD blank)/(OD control − OD blank)] × 100%.

### 2.10. Hemadsorption Assay

PAMs were seeded in 96-well plates at a density of 2 × 10^5^ cells per well in 100 μL of cell culture medium. The cells were pretreated with Stattic or DMSO for 4 h and subsequently infected with ASFV. The viral titer at each time point was determined using the 50% hemadsorption dose (HAD_50_) assay to calculate the virus growth curve. For the HAD_50_ assay, PAMs were seeded in 96-well plates and infected with 10-fold serial dilutions of ASFV CN/SC/2019 with eight replicates per dilution. Following infection, 20 μL of 1% freshly prepared porcine red blood cells in saline buffer was added to each well. The plates were incubated at 37 °C and monitored daily for hemadsorption (HAD) around infected cells for up to 7 days post-infection (dpi). The assay was conducted in three independent experiments, and HAD_50_ was calculated using the Reed and Muench method [26].

### 2.11. Indirect Immunofluorescence Assay (IFA)

3D4/21 cells were seeded in 24-well plates containing coverslips (Wuhan, WHB-24-CS-10, China) and either infected with ASFV or transfected with the specified plasmid for 24 h. The cells were washed twice with pre-chilled PBS, fixed with 4% paraformaldehyde solution (BBI, E672002-0500, Shanghai, China) at room temperature for 30 min, and blocked with 4% bovine serum albumin (BSA, Sigma Aldrich, A9647, Kankakee, IL, USA) at room temperature for 1 h. Permeabilization was performed using 0.5% Triton X-100 (Sigma–Aldrich, T8787, Burlington, MA, USA) for 10 min, which was followed by three washes with pre-chilled PBS. The cells were then incubated overnight with primary antibodies at the specified concentrations and for 1 h with secondary antibodies in the dark. After three additional washes with pre-chilled PBS, the coverslips were removed, and the cells were mounted using ProLong™ Glass Antifade Mountant (containing NucBlue™ stain) for nuclear visualization. Colocalization was assessed using a confocal fluorescence microscope (Leica, Wetzlar, Germany). The images are representative of three independent experiments. Scale bars: 25 μm.

### 2.12. EdU Incorporation and Detection for Flow Cytometry

Typically, EdU (5-ethynyl-2′-deoxyuridine) was added to parallel cultures growing exponentially to final concentrations at 10 µM for 2 h. 3D4/21 cell pellets (5 × 10^5^ cells) were vigorously resuspended in 200 µL of ice cold 4% formaldehyde in PBS, fixed for a minimum of 30 min, and washed three times in wash buffer. Then, cells were permeabilized by the subsequent addition of 0.5% Triton X-100 and kept on ice for 10 min before being blocked with 5% BSA for 1 h. Cells were incubated with the click reaction buffer for 40 min at 37 °C and protected from light. The detection of EdU-DNA was performed according to the E-Click EdU Cell Proliferation Flow Cytometry Assay Kit (Green, FITC) (Elabscience, E-CK-A370, Wuhan, China) as per the manufacturer’s instructions. For EdU-DNA, FITC staining was used to measure their fluorescence by flow cytometry (CytoFLEX, Brea, CA, USA).

## 3. Results

### 3.1. RNA-Seq and Sequencing Data Quality Analysis

**Figure 1:**
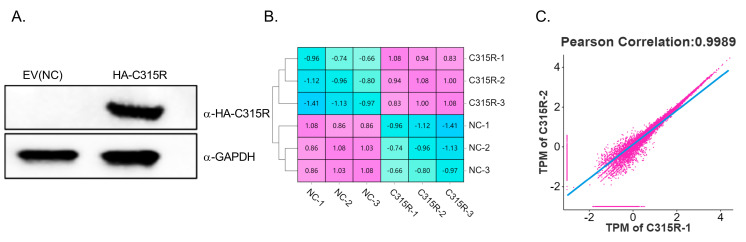
Sample data pattern analysis. (**A**) 3D4/21 cells were transfected with 2 μg of either pcDNA3.1-HA (NC) or pcDNA3.1-HA-C315R (C315R) for 24 h. Successful expression of C315R was confirmed by Western blotting. (**B**) Sample-to-sample distance analysis. The correlation between samples was assessed by listing the identified genes and their respective quantities. A deeper color indicates a high correlation with minimal distance between the samples, while a lighter color reflects a low correlation with greater distance. (**C**) Repeatability scatter plot. The degree of correlation between samples is represented by the proximity of points to the diagonal line with fewer deviations indicating higher correlation.

To investigate the function of C315R, 3D4/21 cells were seeded in 6-well plates and transfected with 2 μg of either pcDNA3.1-HA or pcDNA3.1-HA-C315R for 24 h. Western blotting analysis confirmed the successful expression of HA-C315R in the cells (Figure 1A), and the cell samples were subsequently subjected to RNA-seq analysis. The Pearson correlation coefficient (PCC) was calculated for each pair of samples, and the resulting correlation coefficients were visualized in a heatmap to demonstrate the strong correlation between the samples. The repeatability within each group was further assessed using a scatter plot, where smaller deviations from the diagonal line indicate a higher correlation between the samples (Figure 1B,C).

### 3.2. Differentially Expressed Genes (DEGs)

**Figure 2:**
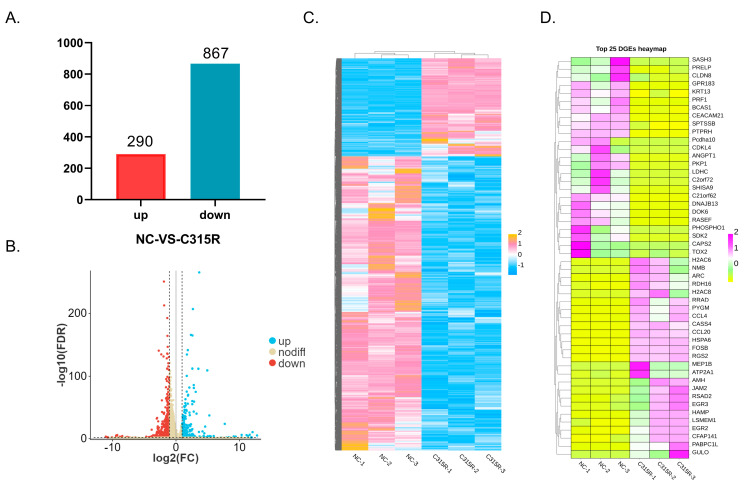
Changes in differentially expressed genes (DEGs) between cells transfected with the plasmid expressing HA-tagged C315R or the empty vector. (**A**) The total number of differentially expressed genes (DEGs) identified in the comparison between NC and C315R groups. (**B**) Volcano plot depicting global DEGs between the NC and C315R samples. Significantly upregulated genes are shown in blue, while downregulated genes are shown in red. (**C**) Heatmap clustering of all DEGs across samples, illustrating the overall expression patterns. Pink and blue colors indicate up- and down- regulation in Log2 cpm values, respectively. Significantly upregulated genes are shown in pink, while downregulated genes are shown in blue. Each column in the graph represents a sample, each row represents a gene, and the expression of genes in different samples is represented by different colors, with pinker colors indicating higher expression and bluer colors indicating lower expressio. (**D**) Heatmap clustering of the top 25 DEGs, highlighting the most significantly altered genes between the NC and C315R groups. Pink and yellow colors indicate up- and down- regulation in Log2 cpm values, respectively. Significantly upregulated genes are shown in pink, while downregulated genes are shown in yellow. Each column in the graph represents a sample, each row represents a gene, and the expression of genes in different samples is represented by different colors, with pinker colors indicating higher expression and yellower colors indicating lower expressio.

Compared to pcDNA3.1-HA-transfected 3D4/21 cells, pcDNA3.1-HA-C315R-transfected 3D4/21 cells exhibited 290 upregulated genes and 867 downregulated genes, totaling 1157 differentially expressed genes (DEGs) (Figure 2A). The fold change (FC) values for each DEG in both groups were calculated using DESeq2 software. To assess the extent of differential expression, a threshold of FDR |Log2FC| ≥ 1 was applied, and the results are visualized in a volcano plot (Figure 2B). The expression profiles of all DEGs were clustered using hierarchical clustering (http://www.heatmapper.ca/expression/ (accessed on 20 December 2024)) (Figure 2C), and a similar clustering analysis was performed for the top 25 DEGs (Figure 2D).

### 3.3. Enrichment Analysis of GO Terms, KEGG Pathways, and Reactome

**Figure 3:**
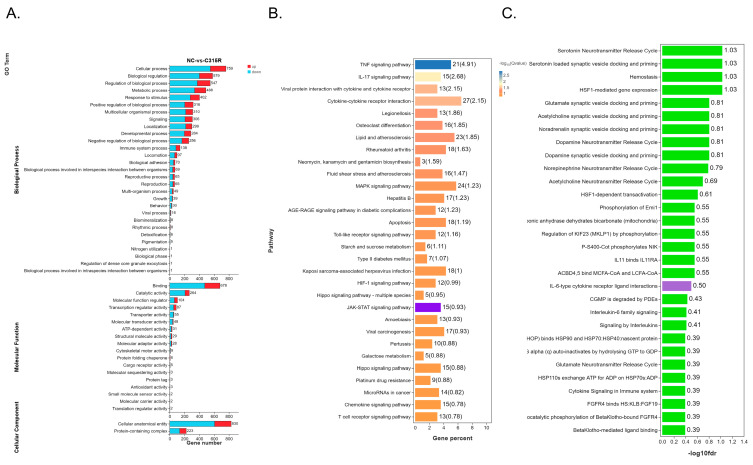
GO, KEGG, and Reactome enrichment of DEGs. (**A**) Bar chart depicting the Gene Ontology (GO) term enrichment of differentially expressed genes (DEGs), categorized into Biological Processes (BPs), Molecular Functions (MFs), and Cellular Components (CCs). (**B**) Bar chart showing the Kyoto Encyclopedia of Genes and Genomes (KEGG) pathway enrichment of DEGs, highlighting significant pathways involved in cellular processes. JAK STAT related factors were significantly enriched. (**C**) Bar chart illustrating Reactome pathway enrichment of DEGs, emphasizing key signaling and regulatory pathways associated with the DEGs. IL-6 related factors were significantly enriched.

After identifying the differentially expressed genes (DEGs), we performed enrichment analyses for Gene Ontology (GO) terms, Kyoto Encyclopedia of Genes and Genomes (KEGG) pathways, and Reactome (*p*-value ≤ 0.05). These analyses provided valuable insights into the functional roles and cellular responses of C315R. The GO terms were classified into three categories: Biological Processes (BPs), Molecular Functions (MFs), and Cellular Components (CCs). The DEGs in the comparison between NC and C315R were primarily associated with cellular processes, the regulation of biological processes, responses to stimuli, transcriptional regulation activity, ATP-dependent activity, and protein-containing complexes (Figure 3A). These findings suggest that C315R plays a crucial role in transcriptional regulation and cellular functions. Next, KEGG enrichment analysis was conducted to explore the signaling pathways associated with the DEGs. The analysis revealed significant enrichment in pathways such as TNF signaling, IL-17 signaling, the JAK STAT signaling pathway, MAPK signaling, and T cell receptor signaling, indicating that C315R plays a pivotal role in modulating inflammatory and immune responses. Additionally, the KEGG analysis highlighted C315R’s involvement in apoptosis, viral protein interactions with cytokines, and cytokine cytokine receptor interactions (Figure 3B). Finally, Reactome enrichment analysis was used to further delineate the functional roles of the DEGs. This analysis showed significant enrichment in pathways such as signaling by interleukins, IL-6-type cytokine receptor ligand interactions, and interleukin-6 family signaling (Figure 3C). Collectively, these enrichment analyses provide a comprehensive understanding of the biological impact and underlying mechanisms of C315R.

### 3.4. Protein Protein Interaction (PPI) Network Analysis of DEGs

**Figure 4:**
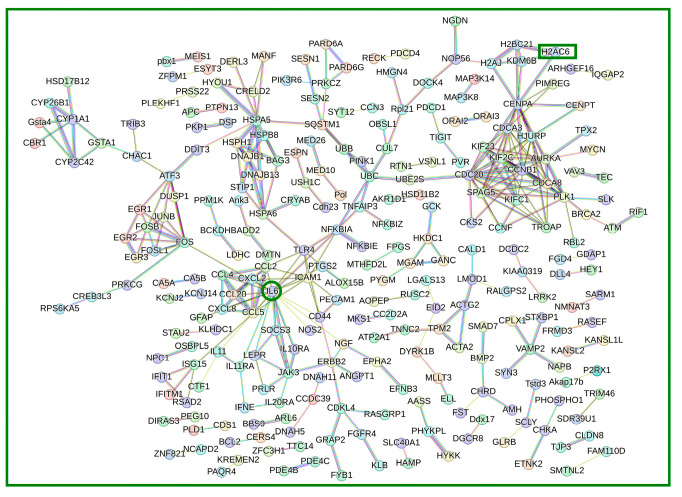
Protein–protein interaction (PPI) network of significant DEGs. The PPI network was constructed using the STRING database to visualize interactions among differentially expressed genes (DEGs) with |Log2FC| ≥ 2 and high confidence (0.70). The network highlights key hub genes, including CDC20, CDCA8, H2AC6, HSPA5, IL-6, CCL2, UBB, and CENPA, which are implicated in cellular responses to chemical stimuli, the regulation of transcription, and DNA damage response, underscoring their potential roles in mediating C315R’s effects on host cell processes, IL-6 and H2AC6 also play important roles in the protein protein interaction network.

To further investigate the biological significance of the differentially expressed genes (DEGs), a protein protein interaction (PPI) network was constructed and visualized using the STRING database (|Log2FC| ≥ 2, High confidence = 0.70). Key DEGs, including CDC20, CDCA8, H2AC6, HSPA5, IL-6, CCL2, UBB, and CENPA, were identified as central players in the PPI network (Figure 4). The DEGs within the PPI network were predominantly associated with cellular responses to chemical stimuli, the positive regulation of biological processes, DNA damage response, the regulation of DNA-templated transcription in response to stress, and the regulation of cell differentiation. These findings highlight the critical roles that these proteins play in cellular processes and stress responses.

### 3.5. Validation of DEGs by RT-qPCR and Immunolocalization

**Figure 5:**
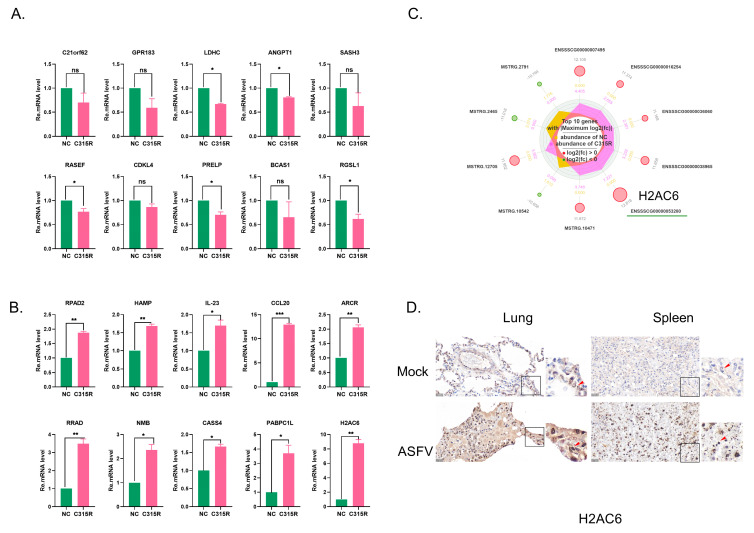
Validation of gene expression differences. (**A**) RT-qPCR validation of significantly upregulated differentially expressed genes (DEGs) between the NC and C315R groups. (**B**) RT-qPCR validation of significantly downregulated DEGs between the NC and C315R groups. Data were shown as mean ± SD based on three independent experiments. Statistical significance is denoted by asterisks (* *p* < 0.05; ** *p* < 0.01; *** *p* < 0.001 determined by two-tailed Student’s *t*-test. ns, no significance). (**C**) KEGG pathway enrichment chord diagram illustrating the top 10 DEGs and their involvement in relevant signaling pathways. (**D**) Immunolocalization images of lung and spleen tissues from ASFV-infected or mock-infected pigs, which was stained for H2AC6. The immunostaining was performed using 5% (*w*/*v*) DAB as a substrate, and all sections were counterstained with hematoxylin to highlight tissue morphology.

To validate the RNA-Seq results, the transcription levels of 20 randomly selected differentially expressed genes (DEGs) were analyzed by quantitative real-time PCR (RT-qPCR) (Figure 5A,B). One of the key genes is H2A clustered histone 6 (H2AC6), a core histone of nucleosome structures, which plays a crucial role in chromatin structure and gene regulation [27]. In our study, C315R overexpression led to a significant increase in H2AC6 transcription, as observed in RNA-seq data, suggesting that ASFV may manipulate the host chromatin structure to facilitate viral replication.

Furthermore, we found that in the C315R group, H2AC6 was significantly upregulated compared to the NC group, as shown in the Reactome enrichment circle diagram (Figure 5C). To further investigate whether H2AC6 expression was upregulated by ASFV infection, immunolocalization was performed. The results showed that H2AC6 expression was notably higher in the lungs and spleens of the ASFV-infected group compared to the mock-infected group (Figure 5D). These findings collectively highlight the potential role of C315R in modulating host transcriptional and signaling pathways, warranting further mechanistic studies. Investigating H2AC6 further could provide insights into ASFV’s strategy for hijacking host nuclear processes.

### 3.6. ASFV Promotes IL-6 Expression to Activate STAT3 Phosphorylation Through C315R

**Figure 6:**
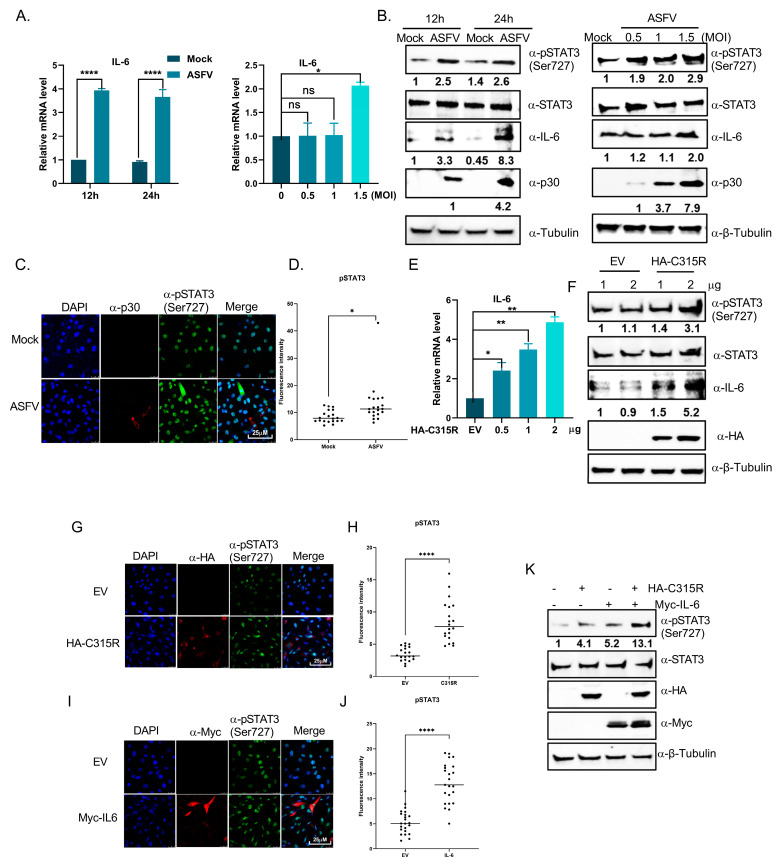
ASFV promotes IL-6 expression to activate STAT3 phosphorylation by C315R. (**A**) RT-PCR analysis of IL-6 mRNA expression in 3D4/21 cells infected with ASFV of different MOIs at different time points post infection, calculated using the 2^−ΔΔCT^ method. The data were analyzed using GraphPad Prism 8.0.2 software. The data were shown as mean ± SD based on three independent experiments. Statistical significance is denoted by asterisks (* *p* < 0.05; ** *p* < 0.01; **** *p* < 0.0001 determined by two-tailed Student’s *t*-test. ns, no significance). (**B**) Immunoblotting of whole cell lysates from 3D4/21 cells infected with ASFV at different time points and MOIs, probing for pSTAT3 (Ser727), STAT3, IL-6, p30, p72, and the cytosolic markers *α-β*-tubulin. (**C**) Confocal microscopy of 3D4/21 cells infected with ASFV at 24 hpi, showing colocalization of p30 (red) and pSTAT3 (Ser727) (green). Nuclei were counterstained with DAPI (blue). Scale bar: 25 μm. (**D**) Fluorescence intensity analysis of individual cells in C. (**E**) RT-PCR analysis of IL-6 expression in 3D4/21 cells transfected with either the empty vector or pcDNA3.1-HA-C315R, calculated by the 2^−ΔΔCT^ method. (**F**) Immunoblotting of lysates from 3D4/21 cells transfected with pcDNA3.1-HA-C315R, probing for pSTAT3 (Ser727), STAT3, IL-6, HA, and *α-β*-tubulin. (**G**) Confocal microscopy showing colocalization of HA-tag (red) and pSTAT3 (Ser727) (green) in 3D4/21 cells transfected with pcDNA3.1-HA-C315R. Nuclei were counterstained with DAPI (blue). Scale bar: 25 μm. (**H**) Fluorescence intensity analysis of individual cells in G. (**I**) Confocal microscopy showing colocalization of Myc-tag (red) and pSTAT3 (Ser727) (green) in 3D4/21 cells transfected with pcDNA3.1-Myc-IL-6. Nuclei were counterstained with DAPI (blue). Scale bar: 25 μm. (**J**) Fluorescence intensity analysis of individual cells in I. (**K**) Immunoblotting of 3D4/21 cells transfected with EV, C315R, IL-6, or C315R/IL-6 co-transfection, analyzing the expression of pSTAT3 (Ser727), STAT3, Myc, HA, and *α- β*-tubulin.

We previously observed significant differences in the IL-6 and JAK STAT signaling pathways between the two groups (Figure 3C). Previous studies have shown that activation of the IL-6 signaling axis induces the autocrine and paracrine phosphorylation of STAT3 in HPV-positive cervical cancer cells, where this pathway is crucial for cancer cell proliferation and survival [20]. Building on these findings, we analyzed the phosphorylation level of STAT3 and IL-6 expression in ASFV-infected cells to determine whether ASFV enhances STAT3 phosphorylation and IL-6 production. Cells were infected with ASFV for varying durations and MOIs, which was followed by RT-PCR and immunoblotting. ASFV-infected cells exhibited significantly higher levels of IL-6 compared to mock-infected cells (Figure 6A). The overall abundance of phosphorylated STAT3 (Ser727) and IL-6 was also increased in ASFV-positive cells compared to ASFV-negative 3D4/21 cells (Figure 6B). Furthermore, immunofluorescence analysis confirmed an increase in the pSTAT3 (Ser727) expression in ASFV-infected cells, which correlated with elevated levels of P30 (Figure 6C,D). These findings collectively demonstrate that ASFV infection leads to increased pSTAT3 (Ser727) and IL-6 expression.

To explore whether C315R modulates IL-6 transcription, RT-PCR was performed, showing that IL-6 expression was upregulated with increasing levels of C315R (Figure 6E). Additionally, 3D4/21 cells were transfected with C315R or an empty vector (EV) control. Results showed that C315R upregulated both pSTAT3 (Ser727) and IL-6 expression (Figure 6F). Immunofluorescence analysis further revealed enhanced pSTAT3 (Ser727) expression in both C315R-overexpressing and IL-6-overexpressing cells (Figure 6G–J). Finally, the co-transduction of C315R and IL-6 in 3D4/21 cells resulted in a significant increase in both pSTAT3 (Ser727) and IL-6 expression compared to other groups, suggesting that ASFV promotes IL-6 expression to activate STAT3 phosphorylation through C315R (Figure 6K).

### 3.7. Inhibition of STAT3 Phosphorylation Reduces ASFV Replication and DNA Synthesis

**Figure 7:**
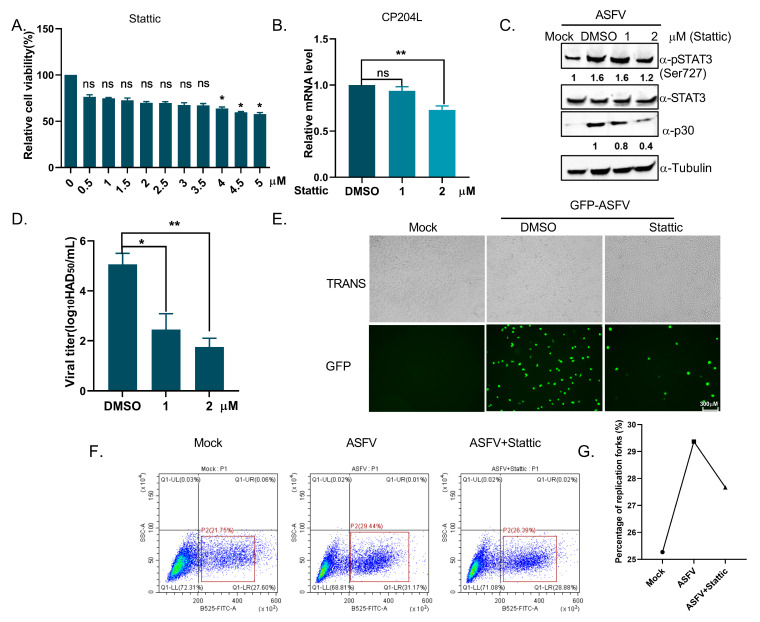
Inhibition of STAT3 phosphorylation reduces ASFV replication and DNA synthesis. (**A**) Cytotoxicity of Stattic was assessed using the CCK8 assay, which was represented as the average of three independent experiments. The data were analyzed using GraphPad Prism 8.0.2 software. (**B**) 3D4/21 cells were pretreated with Stattic or DMSO (control) for 4 h, followed by ASFV infection. IL-6 and p30 mRNA levels were analyzed by RT-PCR and calculated using the 2^−ΔΔCT^ method. Data were shown as mean ± SD based on three independent experiments. Statistical significance is denoted by asterisks (* *p* < 0.05; ** *p* < 0.01 determined by two-tailed Student’s *t*-test. ns, no significance). (**C**) Immunoblotting of 3D4/21 cell lysates treated with Stattic or DMSO for 4 h, probing for pSTAT3 (Ser727), STAT3, p30, and the cytosolic marker *β*-Tubulin. (**D**) PAMs pretreated with Stattic and infected with ASFV showed a significant reduction in viral yields from 5.37 to 2.00 HAD_50_/mL. The data were analyzed using GraphPad Prism 8.0.2 software. (**E**) 3D4/21 cells pretreated with Stattic or DMSO were infected with GFP-ASFV (green) for 24 h. Fluorescence images were captured using a fluorescence microscope (scale bar: 300 μm). (**F**) 3D4/21 cells, mock-infected, ASFV-infected, or ASFV-infected with Stattic for 12 h, were pulsed with 10 μM EdU for 2 h. DNA synthesis was measured by flow cytometry using a 488 nm laser for EdU detection. (**G**) The data from (**F**) were analyzed using GraphPad Prism 8.0.2 software.

To further investigate the role of STAT3 phosphorylation in ASFV replication, we selected STAT3 phosphorylation inhibitors (Stattic) that previously have been reported. The cytotoxicity of Stattic was assessed using a CCK-8 assay on PAMs cells, and no significant cytotoxic effects were observed within the tested concentration range (Figure 7A). To explore the impact of STAT3 phosphorylation on ASFV replication, 3D4/21 cells were pretreated with Stattic or dimethyl sulfoxide (DMSO) as a control for 4 h, which was followed by ASFV infection. RT-PCR and Western blotting analysis revealed a significant reduction in ASFV CP240L mRNA level and p30 expression level in Stattic-treated cells (Figure 7B,C). Similarly, when PAMs cells were pretreated with Stattic and then infected with ASFV, viral yields decreased significantly from 5.37 to 2.00 HAD_50_/mL (Figure 7D). Additionally, GFP-ASFV expression was progressively reduced in Stattic-treated cells (Figure 7E). The phosphorylation of STAT3 is known to be crucial for host DNA transcription. We hypothesize that the inhibition of pSTAT3 impedes viral replication by disrupting DNA synthesis. Thus, we assessed DNA replication levels using an EdU incorporation assay. The results showed that ASFV infection significantly increased DNA synthesis forks, which were reduced by pSTAT3 inhibition, compared to the mock group (Figure 7F,G). These findings suggest that inhibiting STAT3 phosphorylation effectively reduces ASFV replication by impairing DNA synthesis.

## 4. Discussion

ASFV has evolved complex mechanisms to hijack host cellular machinery to promote viral replication, contributing to its ability to persist and spread [28]. The molecular mechanisms underlying ASFV replication and pathogenesis remain incompletely understood despite extensive research on the virus’s ability to exploit host cell machinery for replication [29]. ASFV significantly manipulates the JAK-STAT signaling pathway to modulate immune responses and facilitate its replication. Upon infection, the JAK STAT pathway is typically activated by interferons (IFNs) that trigger the phosphorylation of STAT proteins, leading to the expression of antiviral genes [30]. However, ASFV has evolved several mechanisms to counteract this immune response. For example, ASFV-encoded proteins, such as MGF360-9L, inhibit the activation of STAT1 and STAT2 by promoting their degradation through ubiquitination and proteasomal degradation [31]. This inhibition of the JAK STAT signaling pathway not only suppresses the expression of interferon-stimulated genes (ISGs), which are essential for antiviral defense, but also supports the virus’s ability to replicate efficiently within the host.

Previous studies have highlighted the significance of the JAK STAT pathway in regulating immune responses, particularly in the context of viral infections [32,33]. Our previous study indicated that C315R may induce host translation shutoff, relying on the activation of the PKR/eIF2α pathway and prevent the eIF2α-independent SGs formation, while the over-expression of ASFV C315R significantly reduces most of the cytokine’s expression [34]. In this study, we explored the role of the ASFV C315R protein in modulating host immune pathways, particularly focusing on its impact on IL-6 expression and subsequent STAT3 phosphorylation and its impact on viral replication. Recent studies have shown that ASFV manipulates host immune signaling to promote viral persistence and replication often by modulating key signaling molecules such as IL-6 and STAT3 [35]. Our findings demonstrate that C315R significantly alters the host’s transcriptional landscape, particularly through the regulation of IL-6 expression, leading to the activation of STAT3 phosphorylation, which in turn enhances ASFV replication. Similar mechanisms have been observed in other viral infections, such as HIV and influenza, where the virus-induced activation of STAT3 signaling contributes to viral replication and immune evasion [36,37]. These results further underscore the critical role of the JAK STAT pathway in ASFV pathogenesis and suggest potential targets for therapeutic intervention.

Our RNA-Seq analysis reveals that C315R modulates a wide range of differentially expressed genes (DEGs) associated with various cellular processes, immune responses, and transcriptional regulation. Among the most prominent pathways influenced by C315R are those related to inflammation, immune response, and cellular functions, including TNF signaling, IL-17 signaling, and JAK-STAT signaling (Figure 3). These results suggest that C315R acts as a key regulator in the host immune response, potentially enabling ASFV to manipulate the host cell environment to favor its replication. The observation that C315R affects genes involved in transcription regulation and DNA damage response, such as H2AC6, further supports the notion that ASFV exploits host cellular machinery to drive viral replication. H2AC6 has been shown to play a role in the replication of other viruses, including human papilloma virus (HPV) [38]. In the context of ASFV, our data suggest that the C315R-induced upregulation of H2AC6 may facilitate viral gene transcription and DNA synthesis, which are processes that are essential for efficient viral replication. This finding is in line with previous studies demonstrating that viruses can manipulate host histone modifications to promote their own replication [39].

Additionally, the enrichment of DEGs in pathways such as cytokine cytokine receptor interactions (Figure 3) indicates that C315R influences the cytokine network, which is a hallmark of virus host interactions. This is consistent with reports on other viruses like hepatitis C virus (HCV) and Epstein Barr virus (EBV), which utilize host cytokine signaling to enhance replication and evade immune detection [40,41]. One of the most significant findings of this study is that C315R enhances IL-6 expression, which in turn activates STAT3 phosphorylation (Figure 6A,B). The IL-6/STAT3 pathway is a well-known regulator of immune responses, and its dysregulation is often linked to viral pathogenesis. IL-6 is a potent cytokine that triggers autocrine and paracrine signaling, leading to STAT3 activation, which is a key player in cell survival, proliferation, and immune regulation [42]. The ability of C315R to upregulate IL-6 expression and enhance STAT3 phosphorylation aligns with previous studies on other viral infections. For example, in the case of human papilloma virus (HPV), the viral E6 protein activates IL-6 production, which then triggers STAT3 activation, contributing to the proliferation of infected cells [20,43]. Our results showing increased IL-6 levels and pSTAT3 expression in ASFV-infected cells suggest that C315R likely plays a similar role in enhancing ASFV replication by manipulating the host’s immune response through IL-6/STAT3 signaling.

Our data indicate that the inhibition of STAT3 phosphorylation significantly impairs ASFV replication and DNA synthesis. Specifically, the STAT3 phosphorylation inhibitor Stattic decreased ASFV replication in both 3D4/21 cells and PAMs, as evidenced by the reduced expression of ASFV markers, such as CP240L mRNA and P30 protein (Figure 7B,C). The reduction in ASFV replication was further confirmed by the decreased viral yields in PAMs pretreated with Stattic (Figure 7D). Additionally, GFP-ASFV expression was notably reduced in Stattic-treated cells (Figure 7E). These results suggest that STAT3 activation plays a crucial role in promoting ASFV replication. STAT3 is known to regulate genes involved in DNA replication and repair, and its phosphorylation can induce the expression of pro-survival factors, thus supporting viral DNA synthesis [44]. In this study, we observed that the inhibition of STAT3 phosphorylation by Stattic also suppressed DNA synthesis, which is critical for viral genome replication (Figure 7F,G). Previous research has demonstrated that STAT3 promotes host DNA replication, and its inhibition can interfere with both viral and cellular DNA synthesis [45]. Inhibiting STAT3 phosphorylation by Stattic in our study significantly impaired ASFV replication, suggesting that STAT3 is an essential factor for ASFV replication in the host cell. These results support the hypothesis that STAT3 phosphorylation contributes to viral replication by maintaining cellular or viral DNA replication processes, thus enhancing the virus’s ability to replicate within host cells. Moreover, the impact of STAT3 inhibition on viral DNA synthesis and replication could provide potential therapeutic targets for ASFV control. As there are currently no specific antiviral drugs for ASFV, targeting key host cell signaling pathways like STAT3 could represent a novel strategy for limiting viral replication. This approach has been explored in other viral diseases, where targeting host cell factors critical for viral replication has led to promising antiviral therapies [45,46].

## 5. Conclusions

**Figure 8:**
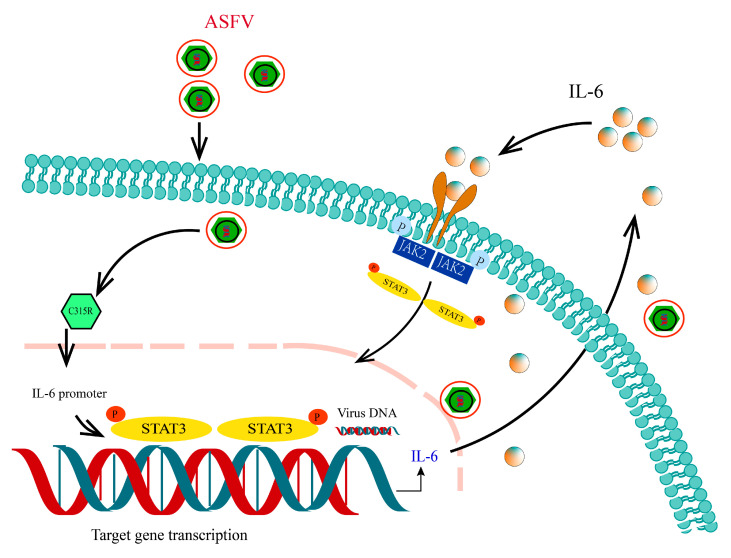
Model of C315R-mediated activation of the IL-6 STAT3 signaling axis facilitating ASFV infection. C315R promotes the transcription of the pro-inflammatory cytokine IL-6, which in turn activates STAT3 phosphorylation (pSTAT3). Phosphorylated STAT3 translocate to the nucleus, where it enhances the transcription of viral genes, thereby supporting ASFV replication.

In summary, our study highlights the central role of the ASFV C315R protein in modulating host cell signaling pathways, particularly the IL-6/STAT3 axis, to enhance viral replication (Figure 8). By upregulating IL-6 and promoting STAT3 phosphorylation, C315R facilitates the viral replication cycle and impairs host immune responses. Our findings not only provide new insights into the molecular mechanisms of ASFV pathogenesis but also suggest potential therapeutic targets for the development of antiviral strategies to combat this devastating disease. Further research into the detailed mechanisms through which STAT3 promotes viral replication, including the specific genes and pathways involved, is needed to fully exploit this therapeutic target.

## Figures and Tables

**Table 1:** The primers used in this study.

GenBank Number	Primers	Sequences
XM_021068249	C21orf62-F:	ggctggcttccttggcgtat
	C21orf62-R:	tgcacaacaggtttgccagg
XM_021065849	GPR183-F:	ccaccgctttgcctacacga
	GPR183-R:	gcaccacagcaatgaagcgg
KU705623	LDHC-F:	aactggtgccgtaggcatgg
	LDHC-R:	gaccagggcaaggcgagttt
NM_213959	ANGPT1-F:	ggtcacactgggacagcagg
	ANGPT1-R:	ggaggggccacaagcatcaa
XM_003135376	SASH3-F:	acacagggcctttctgtggc
	SASH3-R:	ctcgggcagcacatccacat
XM_021064670	RASEF-F:	cgggcttggcaggatttcca
	RASEF-R:	tgccttgtcctgggctctct
XM_021087585	CDKL4-F:	ctcctgacaggccagccact
	CDKL4-R:	ggagctctccaggagttggga
XM_005656649	PRELP-F:	cagctcaacctggcccacaa
	PRELP-R:	ttgttgatggcgggcacact
NM_001110175	BCAS1-F:	atcccacgcttctcccacct
	BCAS1-R:	ggctgtcacctggcaccttt
XM_021063761	RGSL1-F:	cacctccacatggaagcccc
	RGSL1-R:	tgcacaggcatcagcacaca
NM_001123127	HSPA6-F:	ccctgaacccccacaacacc
	HSPA6-R:	tagcatacgcgcaccttggg
NM_213817	RSAD2-F:	gttctgctggctgagggcaa
	RSAD2-R:	agttgacgctggttggggtg
NM_214117	HAMP-F:	cctcctgctcctcctcctcc
	HAMP-R:	agtgggtgtctcgcctcctt
AY948114	IL23-F:	agttcccaggctaggggtcg
	IL23-R:	gtgccatccttgagctgtggt
BX119912	ARCR-F:	ggtgttctaccgcctggagc
	ARCR-R:	gtgatggcgtaggggctgac
AJ577084	CCL20-F:	gctgctactccacctctgcg
	CCL20-R:	tgccgtgtgaagcccacaat
XM_021094029	RRAD-F:	gtgcccatcatcctcgtggg
	RRAD-R:	gtggtgcaatgccgctgatg
NM_001123145	NMB-F:	ggatgttcggcagcctcctg
	NMB-R:	ctcggatcttgctggctcgg
NM_001110431	CASS4-F:	cagagaattccgcgggccat
	CASS4-R:	cgtcggtggatgtggtggag
XM_013985397	PABPC1L-F:	ctccagcccaggctgcatac
	PABPC1L-R:	caactggtggccggaccatt
XM_069564667	H2AC6-F:	ctcgcgccaaagcgaaatcc
	H2AC6-R:	ggtactctaacaccgccgcc
NM_002046	GAPDH-F:	gattccacccatggaaattc
	GAPDH-R:	ctggaagatggtggtgatgggatt

## Data Availability

All the data generated or analyzed during the current study are included in this article.
